# 18-α-glycyrrhetinic acid alleviates oxidative damage in periodontal tissue by modulating the interaction of Cx43 and JNK/NF-κB pathways

**DOI:** 10.3389/fphar.2023.1221053

**Published:** 2023-07-19

**Authors:** Niuben Cao, Xiaomeng Liu, Yubo Hou, Yu Deng, Yu Xin, Xirui Xin, Xinchen Xiang, Xinchan Liu, Weixian Yu

**Affiliations:** ^1^ Department of Periodontics, Hospital of Stomatology, Jilin University, Changchun, China; ^2^ Department of Dental Implantology, Hospital of Stomatology, Jilin University, Changchun, China; ^3^ Department of Geriatric Stomatology, Hospital of Stomatology, Jilin University, Changchun, China; ^4^ Jilin Provincial Key Laboratory of Tooth Development and Bone Remodeling, Changchun, China

**Keywords:** periodontitis, 18-α-glycyrrhetinic acid, oxidative stress, apoptosis, gap junctions, connexin43

## Abstract

**Objective:** Periodontitis is a common chronic inflammatory disease in which oxidative stress is one of the key pathogenic factors. Connexin43 (Cx43) is the most critical and widely distributed connexin isoform. When the organism undergoes a severe and sustained stress response, Cx43-mediated gap junctions (GJs) are believed to underlie the biology of tissue injury exacerbation and amplification. Notably, 18-α-glycyrrhetinic acid (GA) is a classical pharmacological inhibitor of GJs and has antioxidant potential. However, the regulatory role of GA in the redox signaling of periodontal tissues and the potential mechanisms of Cx43 in the pathogenesis of periodontitis remain uncertain.

**Methods:** In this study, we evaluated the effects and mechanisms of GA in alleviating oxidative damage of periodontal tissues and cells by constructing an H_2_O_2_-induced oxidative stress model in human periodontal ligament cells (hPDLCs) and a periodontitis model in rats.

**Results:** Cellular experiments showed that GA effectively attenuated H_2_O_2_-induced oxidative damage in hPDLCs by inhibiting the expression and function of Cx43. In addition, pretreatment of hPDLCs with either GA or SP600125 (a JNK inhibitor) inhibited the Cx43/JNK/NF-κB pathway, restored cell viability, and reduced apoptosis. Animal experiment results showed that GA intervention reduced alveolar bone resorption and periodontal tissue destruction, inhibited osteoclast differentiation, improved mitochondrial structural abnormalities and dysfunction in periodontal tissue, and decreased oxidative stress levels and apoptosis in rats with periodontitis.

**Conclusion:** Overall, our findings suggest that the Cx43/JNK/NF-κB pathway may play a vital role to promote periodontitis progression, while GA reduces oxidative stress and apoptosis by inhibiting the interaction of Cx43 and JNK/NF-κB pathways, thus alleviating oxidative damage in the periodontal tissues.

## 1 Introduction

Periodontitis is an inflammatory disease resulting from infection with periodontal pathogenic microorganisms and dysregulation of the host immune system (Ebersole et al., 2020). More than 50% of adults worldwide are affected by different degrees of periodontitis (Eke et al., 2020). Numerous studies found that oxidative stress is a critical pathogenic factor in periodontitis, which is caused by an imbalance between the production of reactive oxygen species (ROS) and the endogenous antioxidant defense system (Ornatowski et al., 2020; Sczepanik et al., 2020). Under physiological conditions, ROS that have antimicrobial and bacteriostatic effects can kill invading pathogens in the body. However, excessive ROS production can exert cytotoxic effects on host cells, including oxidative damage to DNA and proteins, disruption of cell growth, and induction of cell apoptosis (Kanzaki et al., 2017). Therefore, further exploration of oxidative stress-related mechanisms is crucial for the development of effective treatments for periodontitis.

As a common form of intercellular communication, gap junctions (GJs) constitute an efficient network system for transmitting information among cells (Leithe et al., 2018). It is formed by the docking of two hemichannels on the plasma membrane of adjacent cells, creating a direct channel for signal transmission between cells (Boal et al., 2021). GJs allow small signaling molecules to exchange between cells without depending on the extracellular environment, including amino acids, nucleotides, second messengers, and cellular metabolites (Vicario et al., 2017; Totland et al., 2020). This process is referred to as gap junction intercellular communication (GJIC) (Epifantseva and Shaw, 2018). Under physiological conditions, GJs play a crucial role in regulating various cellular activities and maintaining organismal internal environmental homeostasis by transmitting “protective signals” and “death signals” intercellularly (Yuan et al., 2019). However, severe tissue damage may induce when the balance between these two signals is disturbed. Tirosh et al. (2021) discovered that cells in pathological conditions can transmit “death signals” via GJs to neighboring normal cells, leading to damage in the neighboring cells. This phenomenon is known as the “bystander effect (Tirosh et al., 2021).” Therefore, GJs are regarded as the biological basis for the deterioration and amplification of tissue damage (Huang et al., 2019). Moreover, GJs also involved in the transmission of ROS between cells. This process is influenced by various factors, including diffusion rate, half-life, lipid permeability, and intracellular redox status (Qin et al., 2021). It has been reported that increased GJIC function is closely associated with bronchopulmonary dysplasia (BPD) and secondary brain injury (SBI) pathogenesis (Qing et al., 2021; Zhang et al., 2021). Given that oxidative stress is a vital pathogenic factor for BPD and SBI, it suggests that enhanced GJIC function may be a common characteristic of diseases where oxidative stress is the primary pathogenic mechanism (Qing et al., 2021; Tirosh et al., 2021).

Connexins are the constituent structures of GJs and are designated with their molecular weight (in kDa) (Totland et al., 2020). Among the 21 connexin isomers identified in the human genome, Connexin43 (Cx43) is the most widely expressed and critical connexin. It plays an essential role in regulating GJs function, controlling cell migration and proliferation, wound repair, and immune regulation (Turovsky et al., 2021; Niemann et al., 2022). In 2013, Kato et al. first demonstrated the existence of functional GJs in hPDLCs (Kato et al., 2013). Subsequently, Wu et al. found that Cx43 was the highest expression level among the connexin family in hPDLCs (Wu et al., 2022). Remarkably, it has been reported that Cx43 may be a key intermediate connecting oxidative stress and inflammation to cellular damage, and targeting Cx43 could potentially be a therapeutic approach for certain inflammatory diseases (Huang et al., 2019). However, the role of Cx43-mediated GJs in periodontitis remains poorly known.

The gating of GJs is regulated by several factors, including changes in pH, intracellular ion concentration, and transmembrane potential (Ai et al., 2021). Furthermore, the C-terminal tail of Cx43, which is located in the cytoplasmic matrix, contains multiple protein interaction sites. These sites are targeted by numerous protein kinases that regulate the opening and closing of Cx43 channels (Totland et al., 2020). C-Jun N-terminal kinase (JNK) mediates fundamental biological processes in response to external stress signals and is considered an essential regulator of inflammatory responses and immune responses (Chen et al., 2018). The nuclear factor kappa-B (NF-κB), a downstream signaling factor of JNK, plays a significant role in transducing apoptosis and inflammatory signals (Himaya et al., 2012; Kaltschmidt et al., 2021). It has been reported that the JNK/NF-κB pathway is critical to promote osteoclast differentiation and inflammatory responses in periodontal tissues (Chen et al., 2020; Sun M. et al., 2020). More importantly, JNK signaling molecules are involved in regulating the expression of Cx43 in hPDLCs (Wu et al., 2022). However, the regulatory relationship between Cx43 and the JNK/NF-κB pathway, as well as its role in periodontitis progression, are still not fully elucidated.

18-α-Glycyrrhetinic acid (GA) is a triterpenoid compound that exhibits a diverse range of pharmacological activities, including anti-inflammatory and anti-aging effects (Li et al., 2017; Lefaki et al., 2020). Zhang et al. (2022) found that GA effectively alleviated pulmonary inflammation and fibrosis in mice. More importantly, GA is also a classical pharmacological inhibitor of GJs and is widely used in gap junction-related studies (Zhou et al., 2021). Akutagawa et al. (2019) found that topical application of GA in periodontal pockets of mice with periodontitis significantly reduced the levels of inflammatory factors in periodontal tissue and serum, ultimately attenuating the inflammatory response in gingival tissue. However, the specific mechanism underlying how GA modulates the redox state in periodontal tissues remains unknown.

Therefore, this study aimed to investigate the regulatory role of GA on redox signaling in periodontal tissues, as well as the interconnection of oxidative stress, Cx43, and the JNK/NF-κB pathway in periodontitis pathogenesis.

## 2 Materials and methods

### 2.1 Experimental ethics

In this study, cell experiments were performed in accordance with the Declaration of Helsinki 1975 (revised in 2013) and approved by the Medical Ethics Committee of Jilin University Dental Hospital (No. 202232). The animal experiments were carried out in strict accordance with the Guide for the Care and Use of Laboratory Animals and were approved by the Animal Ethics Committee of Jilin University (SY202207100).

### 2.2 Cell culture

With informed consent obtained from their legal guardians, patients ranging from 10 to 20 years of age donated third molars. Patient selection criteria included having healthy periodontal tissues, being non-smokers, and having no history of chronic or infectious diseases. All patients have signed an informed consent form.

Isolation and culturing of human periodontal ligament cells (hPDLCs) were performed as previously reported (Ying et al., 2022). Cells at the 3rd-6th passage were used for further treatment.

### 2.3 Cell treatments

To simulate the oxidative stress microenvironment of periodontitis, hydrogen peroxide (H_2_O_2_, Sigma-Aldrich, United States), known as the most common inducer of cellular oxidative stress models, was used to stimulate hPDLCs in this study. When each cell was touching an average of three to five adjacent cells, this cell density facilitated the formation of gap junctions (Wu et al., 2019). In this study, hPDLCs were pretreated with 10 μM GA (Sigma-Aldrich, United States) and 10 μM SP600125 (a JNK inhibitor) (MedChemExpress, United States) for 1 h and then cells were stimulated with 200 μM H_2_O_2_ for 6 h. All data were obtained from three independent experiments.

### 2.4 Cell viability detection of hPDLCs

Cell viability was assessed using the Cell Counting Kit-8 (CCK-8) (Invigentech, United States). Following treatment with the drug or H_2_O_2_, hPDLCs were incubated in CCK-8 solution for 3 h. The optical density values were measured at 450 nm with a microplate reader (Bio-TEK, United States).

### 2.5 Detection of oxidative stress

To assess the oxidative stress in intracellular and periodontal tissues, the levels of ROS, malondialdehyde (MDA), and superoxide dismutase (SOD) were detected.

The total intracellular ROS levels were determined with the ROS Assay Kit (Beyotime, China). Briefly, cells were incubated with DCFH-DA reagent for 20 min at 37°C after treatment. The intracellular fluorescence intensity was then examined under a fluorescent inverted microscope (Olympus, Japan).

Mitochondrial-derived ROS levels in periodontal tissue were measured using the Mito SOX Red kit (Thermo Fisher Scientific, United States). The Mito SOX red reagent can be oxidized by superoxide to show red fluorescence and thus assess the ROS content. The staining process was carried out as directed. The Mito SOX red fluorescence of the sections was detected by confocal microscopy.

The SOD typed assay kit (Hydroxylamine method) and MDA assay kit (TBA method) (Nanjing Jiancheng Bioengineering Institute, China) were used to detect intracellular and serum levels of MDA and SOD activity. The experimental procedures were carried out strictly according to the instructions.

### 2.6 Western blot analysis

The methods and procedures for Western blotting were previously reported (Yue et al., 2021). The information for the primary antibodies is as follows: Cx43 (Abcam, UK; 1:1000), P-JNK (Cell Signaling Technology, United States; 1:1000), JNK (Cell Signaling Technology, United States; 1:1000), NF-κB (Cell Signaling Technology, United States; 1:1000), Caspase-3 (Wanleibio, China, 1:2000), Bax (Proteintech, United States; 1:8000), Bcl-2 (Proteintech, United States; 1:1000), β-actin (Proteintech, United States; 1:10000). The grayscale values were analyzed using ImageJ software.

### 2.7 “Parachute” dye-coupling assay

The “parachute” dye-coupling assay was a classical method for detecting GJIC. The methodology of Wu et al. (2019) was used in this study and their wording was partially replicated in the method statement. When the cells reached over 80% confluence in 12-well plates, one well was designated as the donor cell. The donor cells were co-cultured with 5 μM calcein tetraethyl ester (MedChemExpress, United States) for 30 min. Subsequently, the donor cells were trypsin-digested and inoculated onto the surface of the recipient cells at a ratio of 1:150. The donor-recipient cells were then co-cultured for 4 h to allow for the formation of gap junctions between them. The functionality of GJIC was evaluated by counting the number of fluorescent dye-containing receiver cells around each donor cell.

### 2.8 Quantitative real-time polymerase chain reaction (qRT-PCR)

The experimental procedure of qRT-PCR was previously reported (Ying et al., 2022). qRT-PCR was used to detect the expression levels of *Cx43, IL-1β, IL-6,* and *TNF-α* mRNA in hPDLCs and rat periodontal tissue, as well as the expression levels of *NF-κB, Bax, Bcl-2*, and *Caspase3* mRNA in rat periodontal tissue. Information on the primers for the relevant mRNAs is shown in Supplementary Table S1. The 2^-△△Ct^ method was used to calculate the relative gene expression.

### 2.9 Animal models and group allocation

After a 1-week adaptation period, twenty-four male Wister rats (6 weeks old; 200 ± 20 g) were randomly divided into three groups: control group (C-group), periodontitis group (P-group), and periodontitis + GA group (P + GA-group) [intragastrical administered 30 mg/kg/day. Basis of drug dose: In the available reports on liver protection, the safe dose of GA in rats was 5–75 mg/kg/day, and we chose the most commonly used dose of 30 mg/kg/day (Yang et al., 2017)]. The method of constructing the rat periodontitis model was as we reported previously (Li et al., 2022). Briefly, all rats were systematically anesthetized by intraperitoneal injection of 2% pentobarbital sodium. Next, the periodontitis model was induced by ligating orthodontic ligature wires on the cervical part of the maxillary first molars bilaterally in rats. To eliminate the effect of mechanical trauma on periodontal tissues, we also placed ligature wires in the cervical region of the maxillary bilateral first molars of the control rats. However, the ligature wires were removed immediately after placement. The position of the ligature wire was checked daily to ensure that it was located in the cervical region of the tooth. The rats were euthanized after 2 weeks of periodontitis induction for further experimental analysis. The solvent for GA was 0.5% sodium carboxymethyl cellulose (Aladdin, China). To exclude solvent interference, in this experiment, we gave equal doses of sodium carboxymethylcellulose to the control and periodontitis groups.

### 2.10 Examination of clinical periodontal indicators

The periodontal condition was assessed by examining the pocket depth (PD), tooth mobility (TM), and bleeding index (BI) of the maxillary first molars. The methods and scoring criteria for clinical periodontal indicators are shown in Supplementary Table S2 (Yue et al., 2021).

### 2.11 Micro-computed tomography (Micro-CT) analysis

The maxilla of the rats was scanned using a Micro-CT system (Scanco, Switzerland). The system parameters were set to 70 kV, 200 mA, and 300 m exposure time, with a voxel size of 10 μm. In order to analyze alveolar bone resorption, a region of interest consisting of 40 slices from the root bifurcation of the maxillary first molar was selected. Then, bone mineral density (BMD), bone volume/total volume (BV/TV), and trabecular thickness (Tb.Th) were analyzed and calculated. The distance of the cement-enamel junction (CEJ) to the alveolar bone crest (ABC) was measured and averaged using ImageJ software.

### 2.12 Transmission electron microscope

Mitochondrial ultrastructure in the gingival tissue of rat maxillary first molars was observed using transmission electron microscopy (TECNAI SPIRIT, United States). The samples were processed as we previously reported (Li et al., 2022). The tests were performed at an accelerating voltage of 80 kV. Semi-quantitative analysis of mitochondria was performed using ImageJ software.

### 2.13 Paraffin sections

The maxilla was decalcified by placing it in a 10% ethylene diamine tetraacetic acid (EDTA) solution. After decalcification was completed, the samples were routinely dehydrated and embedded in paraffin. Next, the samples were made into 4 μm paraffin sections along the long axis of the tooth. These paraffin sections will be used for H&E, TRAP, and immunohistochemical staining.

### 2.14 Tartrate-resistant acidic phosphatase (TRAP) staining

Osteoclasts were stained using the TRAP staining kit (Solarbio, China). The staining steps were followed strictly as instructed. Multinucleated TRAP-positive cells on the alveolar bone surface around the first molar are considered active osteoclasts.

### 2.15 Immunohistochemistry

Immunohistochemistry was performed to examine Cx43, P-JNK, NF-κB, 8-OHdG, and SOD1 expression levels in periodontal tissues. The methods and procedures of immunohistochemistry were performed as we previously reported (Li et al., 2022). Briefly, the sections were incubated with primary antibodies against Cx43 (1:500), P-JNK (1:200), NF-κB (1:250), 8-OHdG (Abcam, UK; 1:100), and SOD1 (Abcam, UK; 1:500) overnight at 4°C. All sections were stained with Diaminobenzidine (Solarbio, China) as a substrate for color development and counterstained with hematoxylin.

### 2.16 Mitochondrial membrane potential (MMP)

MMP is one of the important parameters reflecting the function of mitochondria. The experimental procedure was carried out strictly according to the instructions of the JC-1 Mitochondrial Membrane Potential Assay Kit (Solarbio, China).

### 2.17 Terminal deoxynucleotidyl transferase dUTP nick end labeling (TUNEL) assays

Detection of apoptotic cells in periodontal tissues with TUNEL kit (Beyotime, China). The staining process was followed strictly as instructed. Cell nuclei were stained with Hoechst reagent (Solarbio, China). Cells that emit green fluorescence were considered TUNEL-positive cells.

### 2.18 Statistical analysis

Statistical analysis was performed using GraphPad Prism 8.0 software. All data are expressed as mean ± standard deviation (SD). An unpaired Student’s t-test analyzed the differences between the two groups, and the differences between multiple groups were analyzed using one-way ANOVA. *p* < 0.05 were considered statistically significant.

## 3 Results

### 3.1 Effect of oxidative stress microenvironment on Cx43 expression and function in hPDLCs

**FIGURE 1:**
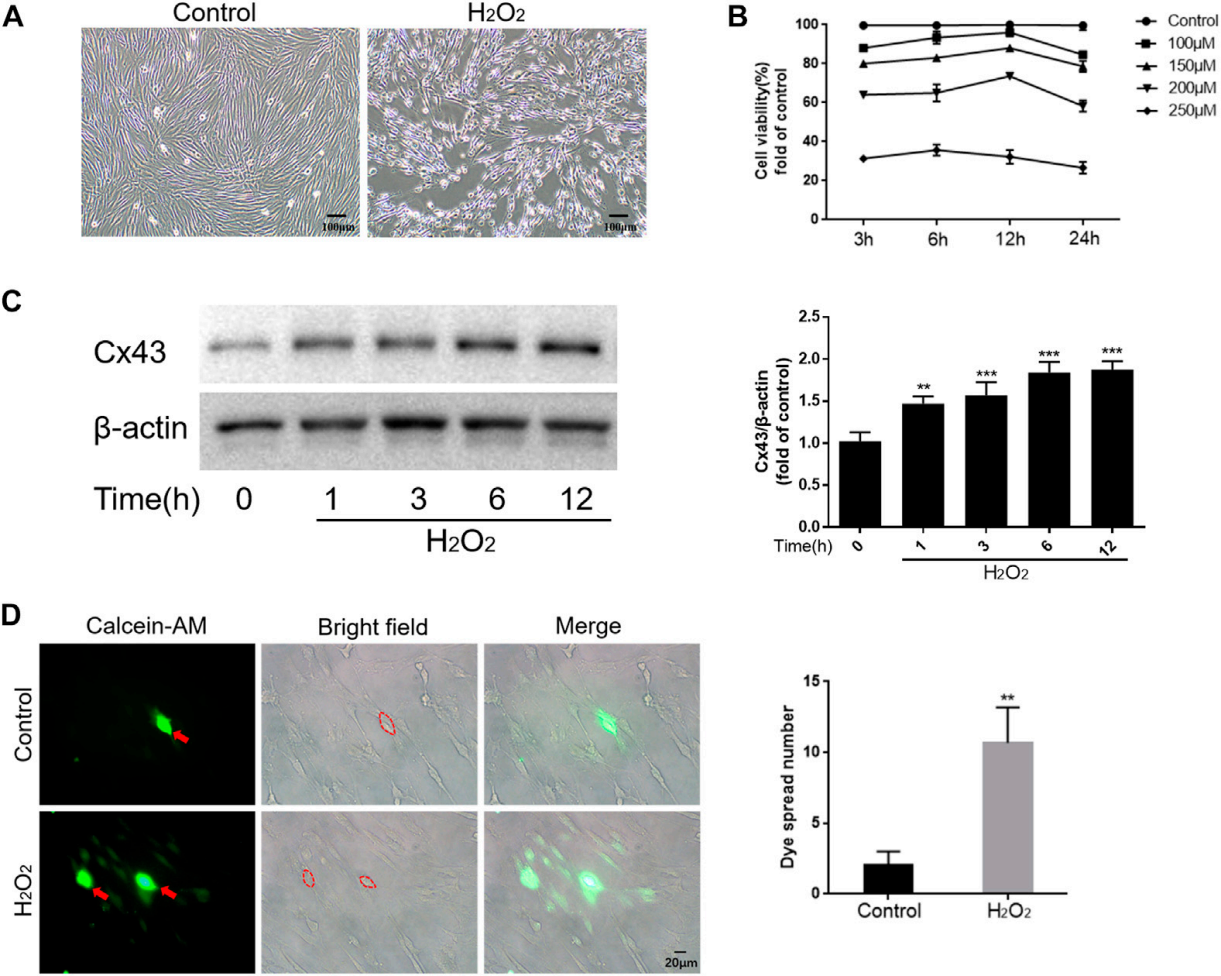
Cx43 may be involved in the oxidative damage of hPDLCs. **(A)** Effect of H_2_O_2_ on the morphology of hPDLCs (Scale bar = 100 μm). **(B)** Altered cell viability. **(C)** Cx43 expression levels were detected at various times under the effect of 200 μM H_2_O_2_ by Western blotting and quantitatively analyzed. **(D)** “Parachute” Dye-Coupling Assay. The ability of calcein tetraethyl ester to diffuse between cells was assayed and quantified in the presence or absence of H_2_O_2_ (200μM, 6 h) treatment (Scale bar = 20 μm). The red arrows and the areas within the dotted line were donor cells. All data were based on three independent experiments and presented as the mean ± SD, **, *p* < 0.01; ***, *p* < 0.001.

To mimic the oxidative stress microenvironment of periodontitis, we treated hPDLCs with different concentrations of H_2_O_2_ for varying durations and then detected the cellular activity. The results showed that H_2_O_2_ reduced cell viability in a dose and time-dependent manner. Notably, when cells were exposed to 200 μM H_2_O_2_, cell viability approached half-inhibitory (Figures 1A, B). Therefore, we determined 200 μM H_2_O_2_ as the optimal concentration for subsequent experiments. Furthermore, we observed a time-dependent increase in Cx43 expression under the effect of H_2_O_2_, peaking at 6 h (*p* < 0.001) (Figure 1C). To further investigate whether increased Cx43 expression correlates with enhanced GJIC function, we performed a “parachute” dye-coupling assay on hPDLCs. Our results showed that the number of receptor cells containing calcein tetraethyl ester was significantly increased under the effect of H_2_O_2_ (*p* < 0.01) (Figure 1D). This suggests a positive correlation between Cx43 expression level and GJIC function, with an enhanced capacity for Cx43-mediated GJIC in hPDLCs under oxidative stress conditions. In summary, we suggest that Cx43 may be engaged in the H_2_O_2_-induced oxidative stress response of hPDLCs.

### 3.2 GA may effectively alleviate H_2_O_2_-induced oxidative damage in hPDLCs by inhibiting Cx43

**FIGURE 2:**
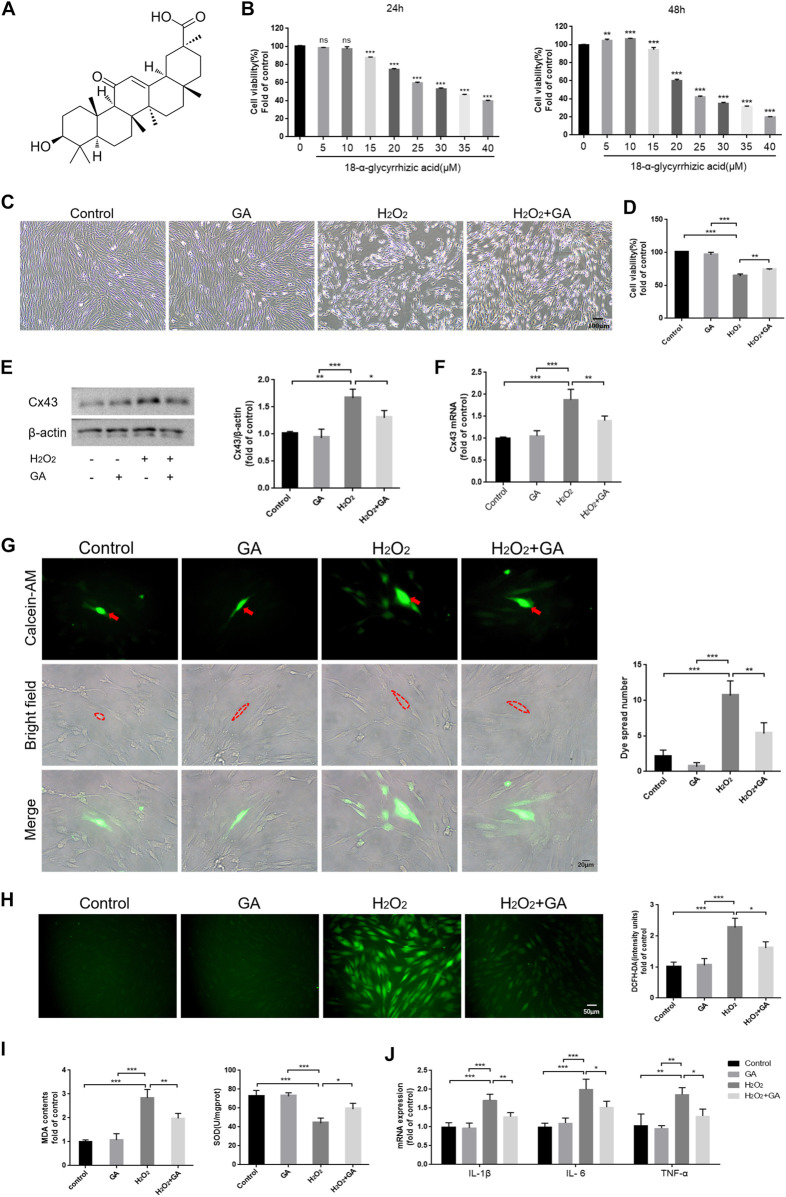
Inhibition of Cx43 may attenuate H_2_O_2_-induced oxidative injury in hPDLCs. **(A)** The chemical structure formula of GA. **(B)** The cytotoxicity of GA. **(C)** Effect of GA on morphological changes of hPDLCs (Scale bar = 100 μm). **(D)** Effect of GA on cell viability. After pretreating hPDLCs with GA (10μM, 1 h) and then stimulating with H_2_O_2_ (200μM, 6 h), cell viability was assayed. **(E)** Cx43 expression in hPDLCs was examined via Western blotting and quantitatively analyzed. **(F)** *Cx43* mRNA expression levels in hPDLCs were detected by qRT-PCR. **(G)** “Parachute” Dye-Coupling Assay (Scale bar = 20 μm). The red arrows and the areas within the dotted line were donor cells. **(H)** DCFH-DA staining results and quantitative analysis in hPDLCs of different groups (Scale bar = 50 μm). **(I)** Detection of MDA levels and SOD activity in hPDLCs. **(J)** The levels of intracellular inflammatory mediator mRNA. All data were based on three independent experiments and presented as the mean ± SD. ns, not significant; *, *p* < 0.05; **, *p* < 0.01; ***, *p* < 0.001.

We cultured hPDLCs with different concentrations of GA (0–40 μM) for 24 h and 48 h to assay the cytotoxicity of GA. The results demonstrated that cell viability was significantly inhibited when the GA concentration exceeded 10 μM (*p* < 0.01) (Figures 2A, B). Therefore, we selected 10 μM GA as the optimum concentration for subsequent experiments.

Following pretreatment of hPDLCs with GA (10 μM, 1 h), we found that cell viability was increased in the GA + H_2_O_2_ group compared to the H_2_O_2_ group (*p* < 0.01), indicating that GA could restore cell viability and promote cell proliferation to some extent (Figures 2C, D). Moreover, the expression level of Cx43 and intercellular communication ability were decreased (*p* < 0.05) in the GA + H_2_O_2_ group, indicating that GA could effectively inhibit the expression and function of Cx43 (Figures 2E–G). Notably, compared to the H_2_O_2_ group, intracellular ROS, MDA levels, and mRNA expression of inflammatory mediators decreased in hPDLCs after GA pretreatment, while SOD activity increased (*p* < 0.05) (Figures 2H–J). In conclusion, GA may effectively alleviate H_2_O_2_-induced oxidative damage in hPDLCs by inhibiting the expression and function of Cx43.

### 3.3 GA may regulate the redox state and apoptosis of hPDLCs by inhibiting the JNK/NF-κB pathway interaction with Cx43

**FIGURE 3:**
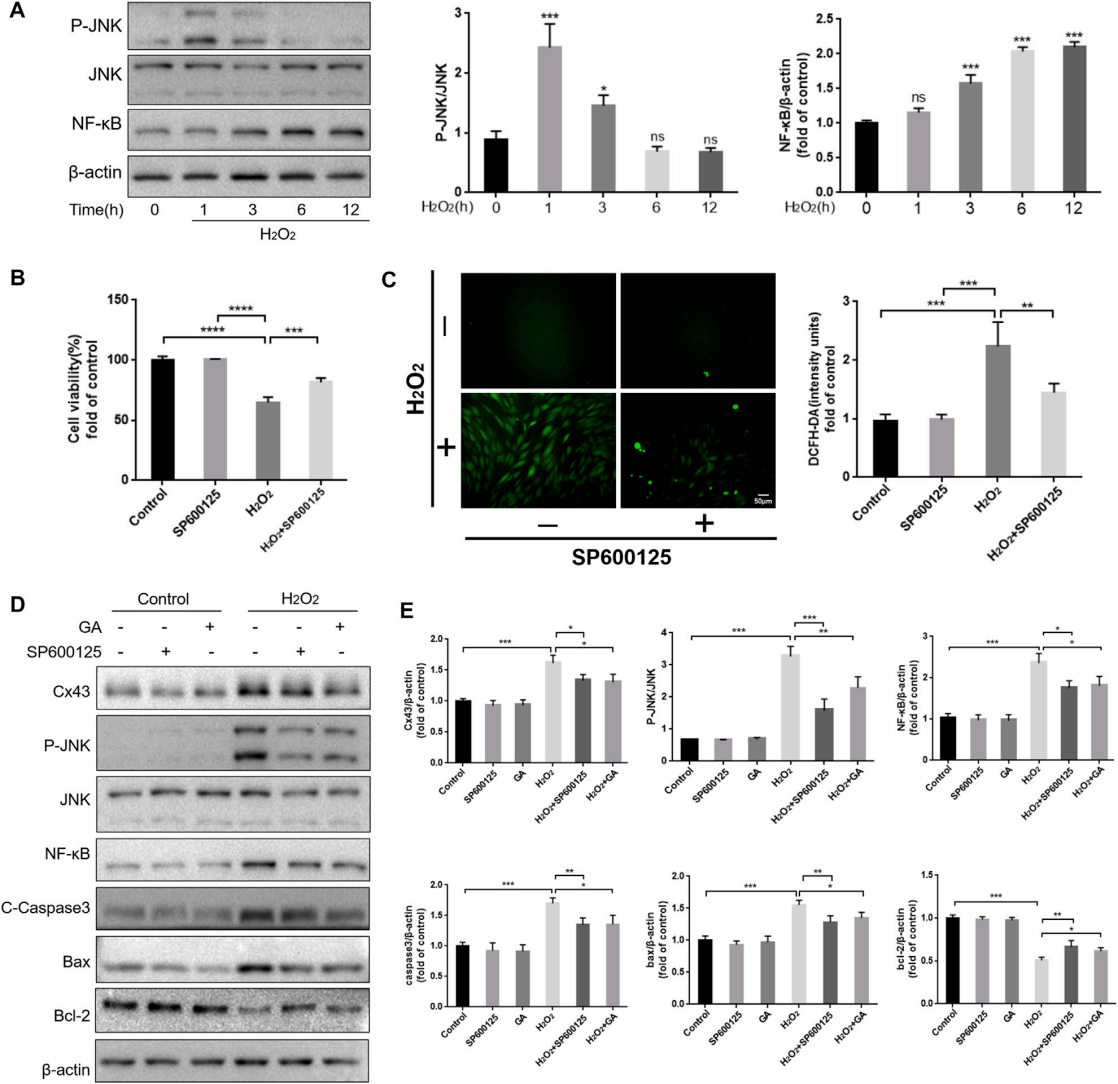
Reciprocal modulation of Cx43 and JNK/NF-κB pathway. **(A)** The JNK/NF-κB pathway may participate in hPDLCs oxidative damage. HPDLCs were stimulated with 200 μM H_2_O_2_ for 0, 1, 3, 6, and 12 h. The JNK and NF-κB expression in cell lysates was detected by Western blotting and quantitatively analyzed. **(B)** Effect of SP600125 on cell viability. **(C)** DCFH-DA staining results and quantitative analysis in hPDLCs (Scale bar = 50 μm). **(D, E)** Cx43 interacts with the JNK/NF-κB signaling pathway to co-regulate apoptosis. HPDLCs were pretreated with GA (10μM, 1 h) or SP600125 (10μM, 1 h) and then stimulated with H_2_O_2_. The expression of Cx43, phosphorylated JNK, NF-κB, and apoptosis-related factors in cell lysates was detected by Western blotting and quantitatively analyzed. All data were based on three independent experiments and presented as the mean ± SD, ns, not significant; *, *p* < 0.05; **, *p* < 0.01; ***, *p* < 0.001.

ROS are an upstream regulator of the JNK/NF-κB pathway (An et al., 2019). Our results showed that JNK was maximally phosphorylated when hPDLCs were exposed to H_2_O_2_ for 1 h. Simultaneously, the expression level of NF-κB in hPDLCs increased in a time-dependent manner under H_2_O_2_ treatment (Figure 3A). In addition, inhibiting the JNK/NF-κB pathway with SP600125 (10 μM, 1 h) restored cell viability and proliferation capacity (*p* < 0.001), and reduced intracellular ROS content (*p* < 0.01) thereby attenuating H_2_O_2_-induced oxidative damage in hPDLCs (Figures 3B, C). These results suggest that the JNK/NF-κB pathway may regulate the redox status within hPDLCs. Given that both the JNK/NF-κB pathway and Cx43 regulate intracellular redox status, we further explored the relationship between the roles of these two factors in the oxidative stress response of hPDLCs. The results showed that compared with the H_2_O_2_ group, the levels of P-JNK and NF-κB were decreased in hPDLCs after pretreatment with GA (10μM, 1 h); consistently, the expression of Cx43 was also decreased in hPDLCs after pretreatment with SP600125 (10μM, 1 h) (*p* < 0.05) (Figures 3D, E), indicating that Cx43 and JNK/NF-κB signaling pathways may interact with each other. Furthermore, under oxidative stress, the expression levels of apoptosis-related factors increased in hPDLCs (*p* < 0.001). However, after pretreatment with GA or SP600125, the expression of the anti-apoptotic mediator Bcl-2 increased, while the expression levels of the pro-apoptotic mediator Bax and the apoptosis executor Caspase3 decreased (*p* < 0.05) (Figures 3D, E). Consequently, we suggest Cx43 may interact with the JNK/NF-κB pathway to co-regulate the redox state and apoptosis of hPDLCs, and GA may alleviate cell damage by inhibiting this interaction.

### 3.4 GA may effectively alleviate periodontitis in rats

**FIGURE 4:**
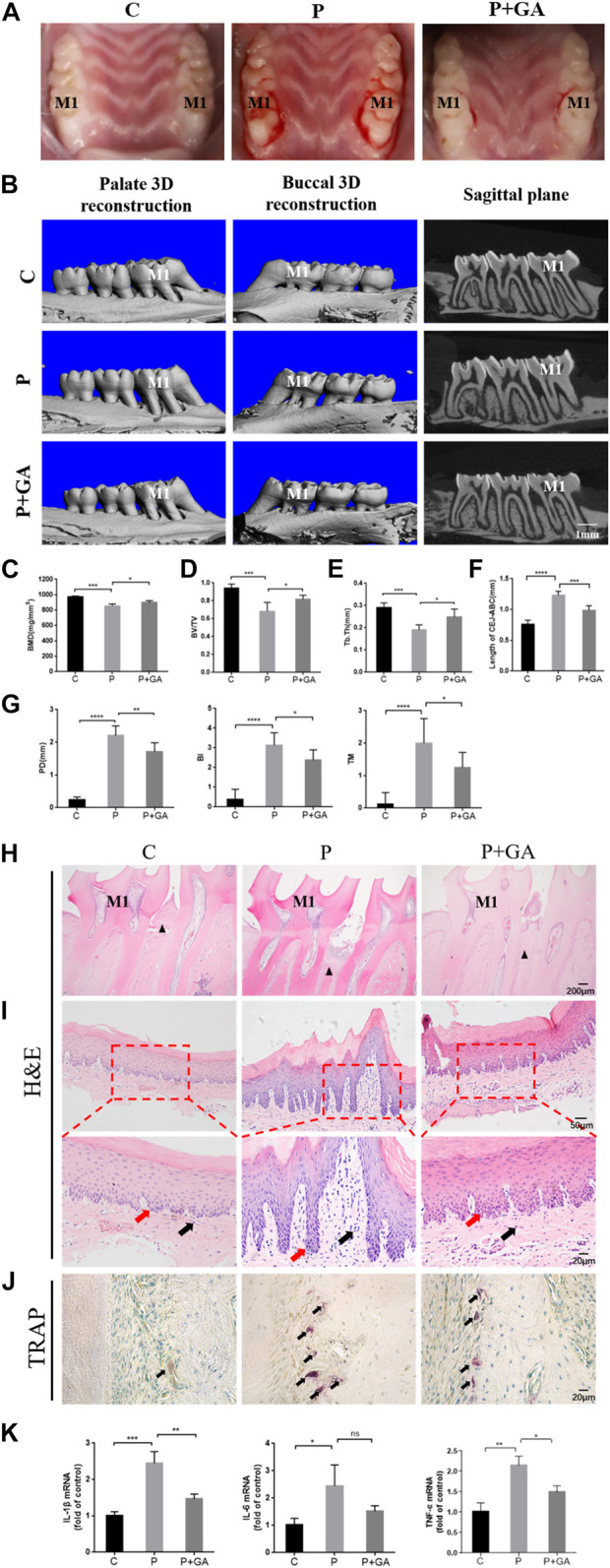
Effect of GA on periodontitis in rats. **(A)** Intraoral photographs of rats. M1 indicates the maxillary first molar. **(B)** Micro-CT scan results (Scale bar = 1 mm). M1 indicates the maxillary first molar. **(C)** BMD (n = 5). **(D)** BV/TV (n = 5). **(E)** Tb. Th (n = 5). **(F)** The distance of CEJ to ABC (n = 5). **(G)** The results of periodontal clinical indices include PD, TM, and BI (n = 8). **(H)** H&E staining results of maxillary first molars (Scale bar = 200 μm). M1 indicates the maxillary first molar. The black triangles indicate the alveolar bone crest. **(I)** H&E staining results of gingival tissue at ×200 and ×400 magnification. Black arrows show inflammatory cells. Red arrows show the epithelial pegs. **(J)** TRAP staining results (Scale bar = 20 μm). The black arrows indicate osteoclasts. **(K)** Expression levels of inflammatory mediators in periodontal tissues. Data are presented as the mean ± SD. ns, not significant; *, *p* < 0.05; **, *p* < 0.01; ***, *p* < 0.001.

We further demonstrated the clear role of Cx43 in periodontitis by constructing a rat periodontitis model. Visual observation showed that the gingiva of the control group rats appeared pink, thin in shape, with a tough texture, and no bleeding during probing. In the rats with periodontitis, the gingiva exhibited redness, swelling, gingival margin hypertrophy, and a soft texture, with significant bleeding during probing, indicating that the rat periodontitis model was successfully constructed in this study. However, after intervention with GA, the periodontal condition improved to some extent (Figure 4A). Furthermore, Micro-CT results showed that compared to the C-group, BMD, BV/TV, and Tb.Th decreased (*p* < 0.001) in the bifurcation zone of maxillary first molar roots in the P-group rats, while the distance from CEJ to ABC increased (*p* < 0.001), indicating that the P-group rats had severe alveolar bone resorption (Figures 4B–F). The periodontal clinical index of the rats in the P-group was higher than the C-group (*p* < 0.001) (Figure 4G). In contrast, the level of bone resorption and clinical periodontal indicators were reduced after the GA intervention (*p* < 0.05) (Figures 4B–G). H&E staining of periodontal tissues showed severe attachment loss and the epithelial pegs grew significantly with massive infiltration of inflammatory cells in the P-group. Additionally, TRAP staining showed that the number of activated osteoclasts on the alveolar bone surface of the P-group rats was significantly increased, further indicating enhanced bone resorption in the alveolar bone. However, the above pathological manifestations were improved in the P + GA-group (Figures 4H–J). Furthermore, we also observed that inflammatory factor expression in periodontal tissues was reduced after GA intervention (Figure 4K). The above results suggest that GA may effectively alleviate periodontitis in rats.

### 3.5 The Cx43/JNK/NF-κB pathway may have a vital role in modulating the periodontitis progression

**FIGURE 5:**
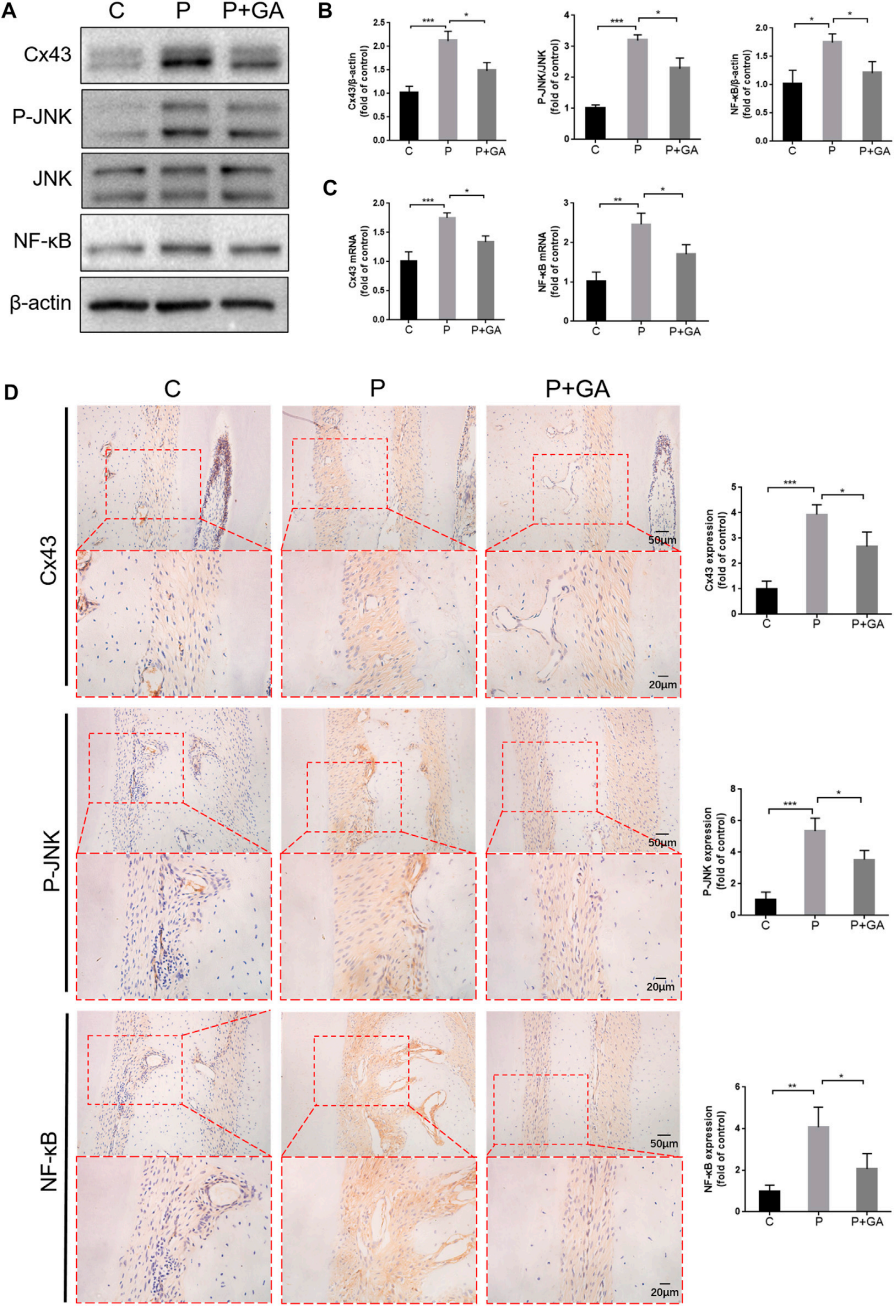
Inhibiting the Cx43/JNK/NF-κB pathway may hinder periodontitis progression. **(A)** Results of protein expression levels of Cx43, P-JNK, JNK, and NF-κB in rat periodontal tissues. **(B)** Quantitative analysis of grayscale values. **(C)** Results of *Cx43* and *NF-κB* mRNA levels in rat periodontal tissues. **(D)** Immunohistochemical staining of Cx43, P-JNK, and NF-κB in rat first molar periodontal tissue at ×200 and ×400 magnification. Data are presented as the mean ± SD (n = 3). *, *p* < 0.05; **, *p* < 0.01; ***, *p* < 0.001.

In view of the significant effect of Cx43 and the interaction between the JNK/NF-κB pathway in H_2_O_2_-induced oxidative damage in hPDLCs, we further detected the role of the Cx43/JNK/NF-κB pathway in periodontitis. The results showed that the expression levels of Cx43 (*p* < 0.001), P-JNK (*p* < 0.001), and NF-κB (*p* < 0.05) were significantly increased in the periodontal tissues of the P-group rats. However, the activation of this signaling pathway in periodontitis can be inhibited after GA intervention (*p* < 0.05) (Figure 5). Consequently, we concluded that the Cx43/JNK/NF-κB pathway may have a critical role in promoting periodontitis progression, while inhibition of this signaling pathway may effectively alleviate periodontitis.

### 3.6 GA may attenuate oxidative damage and reduces apoptosis in periodontal tissues by inhibiting the Cx43/JNK/NF-κB pathway

#### 3.6.1 Effect of inhibiting the Cx43/JNK/NF-κB pathway on oxidative stress response within periodontitis

**FIGURE 6:**
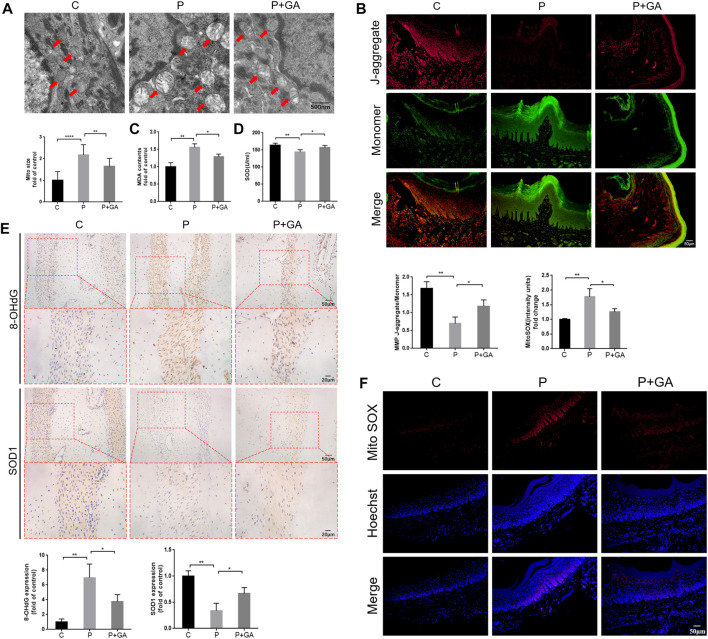
Detection of oxidative stress levels in rat periodontal tissues. **(A)** Transmission electron microscopy scan results and quantitative analysis of mitochondrial surface area (scale bar = 500 nm). Red arrows indicate mitochondria. **(B)** The results of JC-1 staining (scale bar = 50 μm). **(C)** Detection of MDA levels in rat serum. **(D)** Detection of SOD activity in rat serum. **(E)** Immunohistochemical staining of 8-OHdG and SOD1 in rats’ maxillary first molar periodontal tissue at ×200 and ×400 magnification. **(F)** The results of Mito SOX Red staining (Scale bar = 50 μm). Data are presented as the mean ± SD (n = 3). *, *p* < 0.05; **, *p* < 0.01; ***, *p* < 0.001.

As mentioned previously, we found that Cx43/JNK/NF-κB pathway may be involved in regulating intracellular redox status. To confirm this opinion, we examined oxidative stress-related indicators of periodontal tissue in each group. It has been reported that abnormalities in mitochondrial structure and function are closely associated with alterations in intracellular redox status (Sun Y. et al., 2020). Transmission electron microscopy results revealed significant swelling and vacuolization of mitochondria in the gingival tissue of the P-group rats, along with an increase in mitochondrial size (*p* < 0.001); meanwhile, the results of JC-1 staining showed significantly decreased mitochondrial membrane potential in the periodontitis group rats (*p* < 0.01), indicating severe structural changes and dysfunction in the mitochondria of periodontal tissues. However, these alterations were less severe than in the P-group, suggesting that inhibiting the Cx43/JNK/NF-κB pathway improves mitochondrial structural and functional abnormalities in periodontitis (Figures 6A, B). In addition, the levels of ROS content (*p* < 0.01) and 8-OHdG expression (*p* < 0.01) in periodontal tissues, as well as serum MDA levels (*p* < 0.01) were remarkably increased in the P-group compared to the C-group. Conversely, the expression levels of SOD1 (*p* < 0.01) in periodontal tissue and serum SOD activity (*p* < 0.01) were decreased. However, the above indicators were reversed after GA intervention (*p* < 0.05) (Figures 6C–F). The above results suggest that inhibiting the Cx43/JNK/NF-κB pathway may alleviate oxidative stress in periodontitis and thus attenuate oxidative damage in periodontal tissues.

#### 3.6.2 Effect of inhibiting the Cx43/JNK/NF-κB pathway on apoptosis levels within periodontitis

**FIGURE 7:**
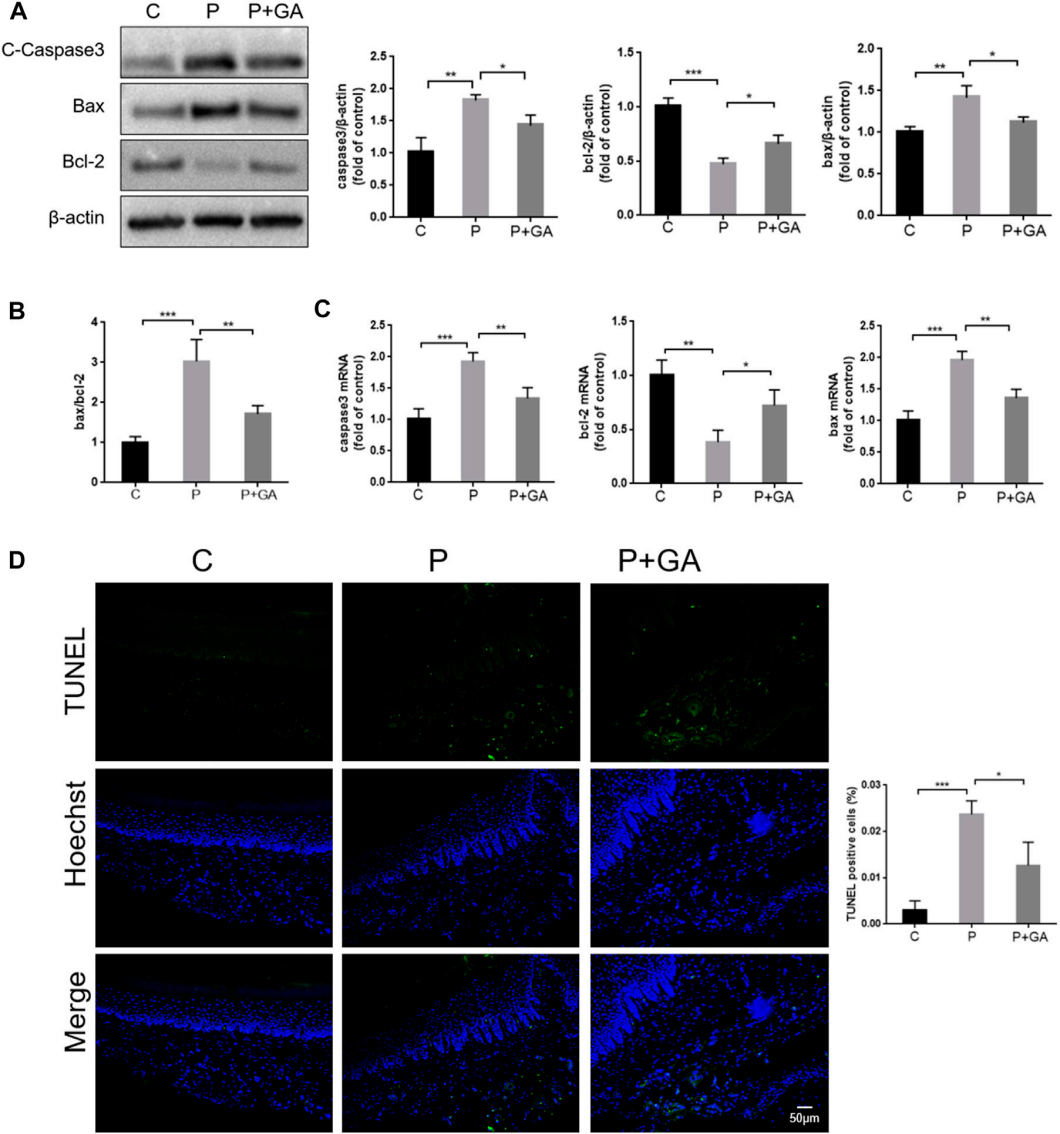
Detection of apoptosis levels in rat periodontal tissues. **(A)** Western blotting examined the expression levels of Caspase3, Bax, and Bcl-2 in the periodontal tissues of rats and quantitative analysis. **(B)** The ratio of Bax/Bcl-2. **(C)** The apoptosis-related factor mRNA expression levels were detected by qRT-PCR. **(D)** TUNEL staining results and semiquantitative analysis (Scale bar = 50 μm). Data are presented as the mean ± SD (n = 3). *, *p* < 0.05; **, *p* < 0.01; ***, *p* < 0.001.

Severe oxidative stress has been reported to induce increased apoptosis, which exacerbates tissue damage (An et al., 2019). As shown in Figure 7, both the gene and protein expression levels of Bax and Caspase3 (*p* < 0.01), the ratio of Bax/Bcl-2 (*p* < 0.001), and the number of TUNEL-positive cells (*p* < 0.001) were increased in the periodontal tissues of the P-group rats compared with the C-group, while the expression level of Bcl-2 was decreased (*p* < 0.01). These findings indicate that increased apoptosis is closely related to the development of periodontitis. However, the expression levels of apoptosis-related factors and the number of TUNEL-positive cells in periodontal tissues were reduced after GA intervention (*p* < 0.05) (Figure 7). In summary, we concluded that inhibiting the Cx43/JNK/NF-κB signaling pathway may attenuate periodontal tissue damage by regulating redox status and reducing apoptosis.

## 4 Discussion

Currently, there is a consensus among numerous scholars that regulating the redox status of periodontal tissues can effectively alleviate periodontitis. However, effective interventions for treating periodontitis are still lacking. In this study, we found that Cx43-mediated GJIC may have an essential effect on the immunomodulation of periodontitis. Meanwhile, we also found that GA may alleviate oxidative stress and reduce apoptosis in periodontal tissues by regulating the interaction of Cx43 with the JNK/NF-κB pathway, which ultimately attenuates periodontal tissue damage. In summary, this research provides a novel therapeutic strategy for treating periodontitis.

Cx43-mediated GJs are considered one of the critical structures for maintaining homeostasis in the organism (Katturajan and Evan Prince, 2021). It not only allows the transport of nutrients and other “protective signals” between cells but also transmits and amplifies “death signals.” Yuan et al. (2019) suggested that under pathological conditions, the transmission of “death signals” is considered to be the main mechanism by which Cx43 channels exert their biological effects and predominate in the oxidative stress response. Although there is no clear definition of “death signals,” Zou et al. (2019) hypothesized that ROS likely belong to this category. In this study, we observed increased levels of Cx43 expression and enhanced intercellular communication capacity in hPDLCs when exposed to an oxidative stress environment. However, inhibiting Cx43 expression and function significantly reduced the levels of oxidative stress biomarkers, increased the activity of the antioxidant SOD, and ultimately attenuated cellular damage. In addition, it is noteworthy that oxidative stress is closely related to mitochondrial structural alterations and dysfunction. Tang et al. (2022) found that Cx43 may cause mitochondrial dysfunction by promoting Bax activity. This study found that mitochondria in the gingival tissue of the periodontitis group rats exhibited significant swelling and vacuolization, and the mitochondrial membrane potential was significantly decreased. However, inhibition of Cx43 may alleviate oxidative stress by attenuating abnormalities of mitochondrial structure and function in gingival tissues. Therefore, we suggest that Cx43 may play an important role in regulating the redox status within periodontal tissues. Inhibiting Cx43 may alleviate periodontal tissue oxidative damage by improving mitochondrial dysfunction, reducing ROS production, and enhancing periodontal tissue antioxidant capacity.

Notably, there are obvious variations in the impact of Cx43-mediated GJs on tissues under different pathological conditions. Maes et al. found that genetically defective mice (Cx43^+/−^) exhibited more severe liver injury than wild-type mice (Cx43^+/+^) when subjected to acute hepatotoxic injury induced by overdoses of drugs (Maes et al., 2016). This suggests that Cx43 channels have a protective effect in this process. In contrast, in nonalcoholic fatty liver disease, increased Cx43-mediated CJs allow stress signals to transmit between hepatocytes, promoting structural changes and dysfunction of the liver (Tirosh et al., 2021). In this context, Cx43 channels seem to have a destructive effect on this process. However, these findings are not contradictory. In response to acute or mild stress response, the organism rapidly regulates the intercellular communication network to “dilute” the toxicity of the damaged cells as a whole, which facilitates the survival of damaged cells (known as the “Good Samaritan effect”). Conversely, when the stress response is strong or persistent, the intercellular communication network becomes a medium for spreading and amplifying cytotoxicity, which leads to more cell damage or even death (known as the “bystander effect”) (Tirosh et al., 2021). Periodontitis is a chronic inflammatory disease whose pathogenesis features the latter group of diseases mentioned above. Meanwhile, this study showed that inhibition of Cx43 may effectively alleviate the symptoms of periodontitis and reduce the resorption of alveolar bone, indicating that Cx43-mediated GJs may be the biological basis for the deterioration of periodontitis and the amplification of periodontal tissue damage.

The “death signals” not only directly cause damage to adjacent cells but also indirectly exacerbate the damage by activating related signaling pathways (Yuan et al., 2019). The JNK/NF-κB pathway has a core effect in modulating various cell functions. However, Overactivation of this pathway can disrupt homeostasis in the organism’s internal environment (Sun et al., 2017). In our study, we found JNK/NF-κB pathway regulates oxidative damage in periodontal tissues and cells. This result was not surprising. ROS have been reported as an upstream regulator of the JNK/NF-κB pathway that participates in regulating its activation (An et al., 2019). In addition, the sustained activation of the JNK/NF-κB pathway can exacerbate mitochondrial dysfunction, leading to the increased release of ROS from mitochondria (Sun et al., 2017). Notably, we found that Cx43 and JNK/NF-κB pathways interact to co-regulate the intracellular oxidative stress state. On the one hand, we suggest that Cx43 may participate in activating the JNK/NF-κB pathway. It was discovered that inhibition of Cx43 attenuates inflammasome activation by downregulating JNK signaling molecules (Huang et al., 2019). In addition, Tien et al. (2021) also found that Cx43 downregulation leads to NF-κB inactivation, leading to a decrease in the release of inflammatory mediators. Consistently, the present study suggests that inhibition of Cx43 may attenuate oxidative damage in periodontal tissues via modulating the JNK/NF-κB pathway. On the other hand, we also found inhibiting the JNK/NF-κB pathway may downregulate Cx43 expression in hPDLCs. Collectively, we suggest that Cx43 expression can be upregulated through the ROS pathway and the JNK/NF-κB pathway under oxidative stress states. At this time, Cx43 overexpression not only enhances intercellular communication, promoting the propagation of ROS between cells, but also feeds back and further activates the JNK/NF-κB pathway, thus forming a “vicious circle” that exacerbates cellular injury. This mechanism may be one of the crucial mechanisms of periodontitis continuously deteriorating.

One of the essential mechanisms for maintaining cell homeostasis by Cx43-mediated GJs may be involved in regulating apoptosis (Huang et al., 2001). Apoptosis is a physiological mechanism of the organism that facilitates the maintenance of the epithelial barrier integrity and regulates the local immune response (Ebersole et al., 2021). However, when the organism is exposed to stimuli such as oxidative stress, it can lead to an increase in apoptosis. At this time, since macrophages cannot remove excessive apoptotic cells, the uncleared apoptotic cells can exacerbate the inflammatory response of the organism and ultimately cause tissue damage (An et al., 2019). The Ebersole et al. (2020) study found that the progression of periodontitis is usually associated with an increase in apoptotic cells. In addition, Li et al. (2020) found that inflammation and damage of periodontal tissues could be effectively alleviated by improving the balance of pro- and anti-apoptotic mediators. It follows that periodontitis pathogenesis is strongly associated with altered apoptosis levels. Cx43 may control cell “fate” by delivering pro- and anti-apoptotic signals. Gurbi et al. (2010) found that Bcl-2 is an important target of Cx43-mediated apoptosis. In addition, Sun et al. (2012) suggested that Cx43 interacts directly with Bax and thus initiates the intrinsic apoptotic pathway. Consistent with these findings, Cx43 overexpression caused increased apoptosis in periodontal tissues and cells in our current study. Notably, JNK signaling molecules have pivotal effects within the intrinsic and extrinsic apoptotic pathways (Dhanasekaran and Reddy, 2017). Recently, one research indicates that inhibiting the JNK/NF-κB pathway may reduce tissue damage by modulating the inflammatory response and reducing apoptosis (Mu et al., 2020). We mentioned above that Cx43 may engage in regulating JNK/NF-κB pathway activation. Therefore, we suggest that Cx43 regulates apoptosis in multiple ways, and activating the JNK/NF-κB pathway may be one of the apoptotic pathways mediated by Cx43.

Unfortunately, there are still limitations in this study. Firstly, although 18-α-glycyrrhizic acid is a classical inhibitor of GJs that has been widely used for more than 40 years in the study of various diseases related to gap junctions, it remains a non-specific drug. Our conclusions may be further confirmed in the future by using genetically defective animal models. Secondly, from a clinical point of view, inhibiting Cx43-mediated GJs for alleviating periodontitis may be a viable therapeutic strategy. However, Cx43 channels play critical physiological functions in multiple organs, including the brain, heart, and liver. Therefore, local administration in periodontal tissues to avoid side effects on vital organs may be a safer and more efficient treatment modality.

## 5 Conclusion

**FIGURE 8:**
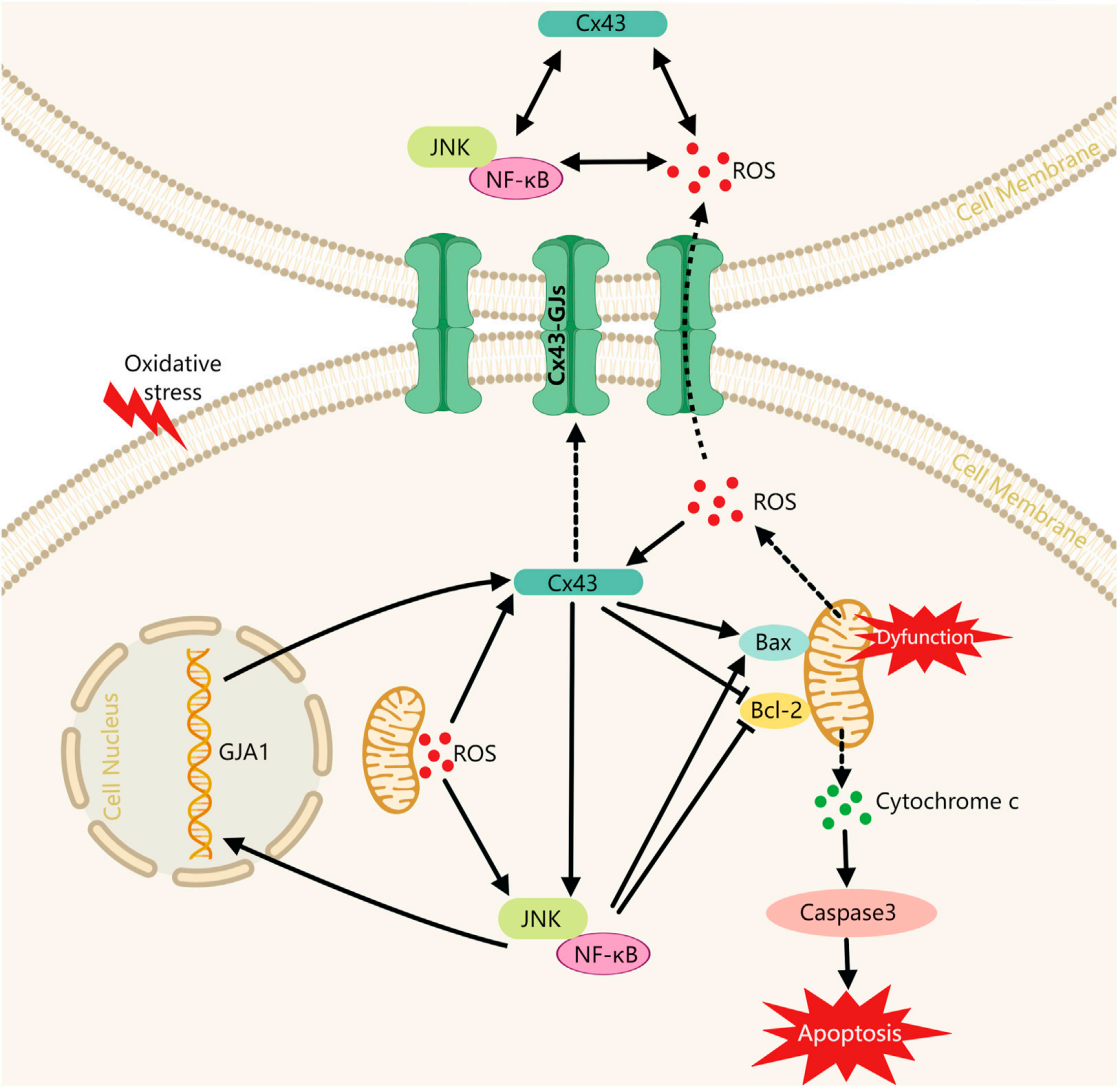
Schematic diagram of Cx43 promoting cell injury via modulating the JNK/NF-κB pathway. Cx43: Connexin43, alias GJA1; Solid lines indicate facilitation or inhibition; dashed lines indicate molecular release and transport processes.

In this study, we have initially revealed the potential role of the Cx43/JNK/NF-κB pathway in periodontitis pathogenesis. The results showed: 1) Cx43 plays a crucial role in regulating oxidative stress response and apoptosis within periodontal tissues, while inhibition of Cx43 by 18-α-glycyrrhizic acid intervention may effectively alleviate periodontitis; 2) Under oxidative stress conditions, Cx43 expression may be upregulated through the ROS pathway and the JNK/NF-κB pathway. The overexpression of Cx43 not only increases intercellular communication capacity but also feeds back and further activates the JNK/NF-κB pathway and forms a “vicious circle.” Overexpression of Cx43 and overactivation of the JNK/NF-κB pathway may promote cell injury. On the one hand, both may exacerbate mitochondrial dysfunction and further promote the release of more ROS from mitochondria. On the other hand, both may inhibit the activity of anti-apoptotic mediator Bcl-2 and promote the expression of pro-apoptotic mediator Bax, which promotes the release of cytochrome C from mitochondria and further activates the apoptotic actuator Caspase3, ultimately leading to apoptosis (Figure 8). Therefore, inhibiting Cx43 or breaking the “vicious cycle” may be a new therapeutic strategy to treat periodontitis.

## Data Availability

The raw data supporting the conclusion of this article will be made available by the authors, without undue reservation.
