# Supplementary material for: 18-α-glycyrrhetinic acid alleviates oxidative damage in periodontal tissue by modulating the interaction of Cx43 and JNK/NF-κB pathways
Source: Front Pharmacol. 2023 Jul 19;14:1221053. doi: 10.3389/fphar.2023.1221053 (PMC10394238; doi:10.3389/fphar.2023.1221053)
Supplement: Supplementary file 1 [file Table2.DOCX]

**Supplementary Materials**

Supplementary Table 1. Primer Sequence Table

| **Gene** | **Gene accession no.** | **Primer sequence (5’-3’)** |
| --- | --- | --- |
| Cx43(GJA1) | NM_000165.5 | F: CTGAGTGCCTGAACTTGCCT |
| (human) |  | R: CTGGGCACCACTCTTTTGC |
| IL-1β | NM_000576.3 | F: GGATATGGAGCAACAAGTGG |
| (human) |  | R: ATGTACCAGTTGGGGAACTG |
| IL-6 | NM_000600.5 | F: GGCGCCTTCGGTCCAGTTGC |
| (human) |  | R: AACCCCATCGCCAAGCCTACCC |
| TNF-α | NM_000594.4 | F: TTGGAGTGATCGGCCCCCAGAG |
| (human) |  | R: TCTCACACCCCACATCTGTCTCCA |
| GAPDH | NM_001256799.3 | F: CGGAGTCAACGGATTTGGTCGTAT |
| (human) |  | R: AGCCTTCTCCATGGTGGTGAAGAC |
| Cx43(GJA1) | NM_012567.2 | F: GTGCTAGCATGGGTGACTGGAGCC |
| (rat) |  | R: GCGGGTACCCAGGATCTCCAGGTCA |
| NF-κB | NM_199267.2 | F: CGACGTATTGCTGTGCCTTC |
| (rat) |  | R: TTGAGATCTGCCCAGGTGGTA |
| IL-1β | NM_031512.2 | F: CAGGATGAGGACCCAAGCAC |
| (rat) |  | R: GTCGTCATCATCCCACGAGT |
| IL-6 | NM_012489.2 | F: CCGGAGAGGAGACTTCACAG |
| (rat) |  | R: CAGAATTGCCATTGCACAAC |
| TNF-α | NM_012675.3 | F: GGCGTGTTCATCCGTTCTC |
| (rat) |  | R: CTTCAGCGTCTCGTGTGTTTCT |
| Bax | NM_017059.2 | F: CCACCAAGAAGCTGAGCGA |
| (rat) |  | R: GCTGCCACACGGAAGAAGA |
| Bcl-2 | NM_016993.2 | F: TTGAGTTCGGTGGGGTCATG |
| (rat) |  | R: GATCCAGGTGTGCAGATGCC |
| Caspase3 | NM_012922.2 | F: AGCCGAAACTCTTCATCATTCA |
| (rat) |  | R: CCATATCATCGTCAGTTCCACT |
| β-actin | NM_031144.3 | F: GGAGATTACTGCCCTGGCTCCTA |
| (rat) |  | R: GACTCATCGTACTCCTGCTTGCTG |

Supplementary Table 2. Methods and scoring criteria of the clinical periodontal indicators.

| **Periodontal clinical indicators** | **Detection methods** | **scoring criteria** |
| --- | --- | --- |
| Pocket depth (PD)  (Unit: mm) | Probing was performed at six sites on the maxillary first molar using a rounded probe (tip radius = 0.2mm), including the mesial site, central site, and distal site on the buccal/palatal side. The probing depth was recorded and averaged. |  |
| Tooth mobility (TM) | Hold the occlusal surface of the maxillary first molar with forceps and gently shake it in all directions. | 0: physiological mobility;  1: slight mobility or buccal-palatal mobility only;  2: moderate mobility or buccal-palatal and medial-distal mobility;  3: severe mobility or buccal-palatal, medial-distal, and vertical mobility. |
| Bleeding index (BI) | The rounded probe was gently probed into the gingival sulcus or periodontal pocket. After removing the probe for the 30s, the presence and extent of bleeding were observed. | 0: normal gingival without inflammation or bleeding;  1: mild inflammation and no bleeding after probe;  2: spotty bleeding after probe;  3: bleeding after probe and blood spreads along the gingival margin;  4: bleeding after probe and blood overflows the gingival crevice;  5: spontaneous bleeding. |
